# Anti-Apoptosis of Podocytes and Pro-Apoptosis of Mesangial Cells for Telmisartan in Alleviating Diabetic Kidney Injury

**DOI:** 10.3389/fphar.2022.876469

**Published:** 2022-04-19

**Authors:** Xin Wei, Yabin Ma, Ya Li, Wenzhao Zhang, Yuting Zhong, Yue Yu, Li-Chao Zhang, Zhibin Wang, Ye Tu

**Affiliations:** ^1^ Department of Clinical Pharmacy, Xinhua Hospital, Shanghai Jiaotong University School of Medicine, Shanghai, China; ^2^ Department of Pharmacy, Shanghai East Hospital, Tongji University, Shanghai, China; ^3^ Department of Clinical Pharmacy, Clinical Trial Center, The First Affiliated Hospital of Shandong First Medical University and Shandong Provincial Qianfoshan Hospital, Jinan, China; ^4^ Department of Critical Care Medicine, School of Anesthesiology, Naval Medical University, Shanghai, China; ^5^ Department of Pharmacy, Shanghai Municipal Hospital of Traditional Chinese Medicine, Shanghai, China; ^6^ Institute of Vascular Disease, Shanghai TCM-Integrated Hospital, Shanghai, China

**Keywords:** diabetic kidney disease, podocytes (MeSH: D050199), mesangial cells, telmisartan (PubChem CID: 65, apoptosis

## Abstract

Podocytes damage and mesangial cells expansion are two important pathological manifestations of glomerular injury in early diabetes. Telmisartan, as an angiotensin type 1 (AT_1_) receptor inhibitor, could improve advanced glycation end (AGE) products or angiotensin Ⅱ (Ang Ⅱ)-induced podocytes injury including detachment or apoptosis. In this current paper, we first confirmed the protective effect of telmisartan on early diabetic kidney injury in type 1 diabetic rats. Telmisartan reduced the loss of podocin and inhibited the expression of α-SMA, reflecting its protective effect on podocyte injury and mesangial proliferation, respectively. More interestingly we observed an opposite effect of telmisartan on the cell viability and apoptosis of podocytes and mesangial cells in a high-glucose environment *in vitro*. The anti-apoptotic effect of telmisartan on podocytes might be related to its inhibition of swiprosin-1 (a protein can mediate high glucose-induced podocyte apoptosis) expression. While telmisartan induced a high expression of PPARγ in mesangial cells, and GW9662 (a PPARγ antagonist) partially inhibited telmisartan-induced apoptosis and reduced viability of mesangial cells. In addition, high glucose-induced PKCβ1/TGFβ1 expression in mesangial cells could be blocked by telmisartan. These data provide a more precise cellular mechanism for revealing the protective effect of telmisartan in diabetic kidney injury.

## Introduction

Diabetic kidney disease (DKD), well known as a chronic kidney disease induced by diabetes mellitus (DM) type 1 or 2 (Podgórski et al., 2019), could worsen glomerular filtration rate (GFR) decreases progressively, then eventually develops into end-stage renal disease (Hou et al., 2018; Ruiz-Ortega et al., 2020; Expert Group of Chinese Society of Nephrology, 2021). There are two mechanisms that hyperglycemia mediate *via* on the kidney are podocytes injury and glomerular basement membrane (GBM) changes induced by mesangial cells expansion or proliferation (Anders et al., 2018).

Podocytes, are specialized visceral epithelial cells, lining the external layer of the GBM, which’s foot processes interdigitate forming an ultimate barrier to prevent urinary protein loss (Podgórski et al., 2019). The number and/or density of each glomerulus have been studied in patients with DM (Papadopoulou-Marketou et al., 2017). Injury to the podocytes contributes to the loss of their adhesive properties and is a major cause of DKD development (Podgórski et al., 2019). Another notable character of podocytes is mature podocytes are limited proliferative cells (Podgórski et al., 2019)^,^ (Griffin et al., 2003). Losses of podocytes bring about proliferation of the mesangial cells, nevertheless more substantial losses lead to glomerular fibrosis and increased proteinuria as subsequent denudation of the GBM (Fukuda et al., 2012). Poor glycemic control results in podocytopathy (Anders et al., 2018), morphological changes characterized by podocytes hypertrophy, podocytes epithelial-mesenchymal transdifferentiation (Chang et al., 2017), podocytes detachment (Zhang et al., 2020), podocytes apoptosis (Wang et al., 2018a) and podocytes loss, which are leading to the progressive podocytes aberrations result in the detachment of the GBM with consequent glomerulosclerosis.

Mesangial cells have a significant impact on not only the adjustment of glomerular and intraglomerular circulation, but also the conservation of glomeruli, such as the defence of glomerular endothelial cells and outflow of substances from serum and fluid from microvessels (Wakisaka et al., 2021). Thickened GBM and expansed mesangial are noticeable glomerular impairments in diabetes (Papadopoulou-Marketou et al., 2017). GBM thickening is an early histopathological in DKD and is affected by the aberrant income and variation of extracellular matrix secreted by endothelial cells and podocytes (Anders et al., 2018). Hyperglycemia excites mesangial cells to proliferate and fabricate matrix (Kriz et al., 2017) *via* activation of transforming growth factor-β (TGFβ), which directly cause the transcriptional activation of matrix collagens (Ziyadeh et al., 2000) conducing to the expanding mesangial matrix.

Early intervention with hypoglycemic and antihypertensive treatment is beneficial to delay the occurrence and development of DKD (Martins and Norris, 2001). Especially recommended in normal blood pressure adults with DM and albuminuria is angiotensin converting enzyme inhibitor (ACEI) or angiotensin receptor blocker (ARB) (Liew et al., 2020). Blockade of the renin–angiotensin system ameliorates the expression of ANGPTL2 and integrin which maintain the glomerular barrier (Tawfik et al., 2021). The reason telmisartan was chosen is that it is described more efficient than other ARB drugs in mitigating proteinuria (Naruse et al., 2019; Guo et al., 2020). Moreover, telmisartan is able to decrease cisplatin-induced nephrotoxicity such as podocytes apoptosis and autophagy-associated protein expression levels (Malik et al., 2015). Fascinatingly, telmisartan has such characters taking into account its twin role of AT_1_ receptor blocking action and peroxisome proliferator-activated receptor gamma (PPARγ) partial agonistic property (Balakumar et al., 2012). The goal of this paper was to examine the protective influence of telmisartan on podocytes injury and mesangial expansion at the early stage of type 1 DM, respectively.

## Materials and Methods

### Animals

SD male rats were purchased from SLRC Laboratory Animal Ltd. (Shanghai, China). Rats were housed at a controlled temperature of 22 ± 2°C, relative humidity of 50–60%, 12-h light and 12-h dark cycles (light, 08:00–20:00, darkness, 20:00–08:00), and allowed free access to standard dry diet and tap water. All animals received humane care, and experimental protocols were approved by the Animal Care Committee at the Naval Medical University.

### Diabetic Model and Treatment

Weight 180–200 g male rats were treated with STZ (Sigma, Deisenhofen, Germany) to induce type 1 diabetes. STZ was dissolved in sterile citrate buffer (pH 4.5) and injected intraperitoneally (65 mg/kg body weight) within 10 min of preparation. The non-diabetic rats initially injected with STZ vehicle served as controls (group Con, *n* = 10). Diabetes mellitus was confirmed by measuring glucose levels tail venous blood using a B-glucose analyzer (HemoCue, Angelholm, Sweden) 7 days later. Rats with random blood glucose level >16.7 mmol/L were included in experiments.

The diabetic rats then received telmisartan (Merck, PHR 1855, 10 mg kg^−1^·d^−1^ po, group DM + Tel, *n* = 10) or vehicle (group DM, *n* = 10) by gavage for 4 weeks. Telmisartan used in this paper was obtained from Sigma-Aldrich Germany, Inc., whose purity is 98%+ (HPLC). Periodically, blood glucose and body weights were measured, and urine samples for quantitative measurement of albuminuria was collected in metabolic cages. Rats were sacrificed under anesthesia after 4 weeks, the kidneys were removed and weighed for histological analysis and protein extraction.

### Urinary Albumin

The urine samples were centrifuged at 10,000 rpm for 5 min to remove insoluble materials. The supernatant was aliquoted and stored at −80°C until used. ELISA kit for rat urinary albumin from Chondrex (Redmond, WA) was used according to the manufacturer’s instructions.

### Creatinine Clearance Rate

Creatinine Assay kit (Nanjing Jiancheng, C011-2-1) was used for the determination of creatinine in blood and urine. Ccr was calculated according to the formula: Ccr = (urinary creatinine*24 h urine volume)/(blood creatinine*24 h*60 min/h)/end weight (kg).

### Immunohistochemical and TUNEL Staining

Kidneys were cut in a slicing microtome at 7–8 μm, and fixed with 4% paraformaldehyde in PBS for 10 min. Blocking has been performed with buffer (PBS, 2% BSA, 10% FBS) for 1 h followed by 10 min incubation with a second buffer (PBS, 0.4% Triton). Primary antibody against *α*-SMA (Servicebio, GB13044) or NPHS2 (Abcam, ab229037) has been incubated for 3 h at room temperature in a humidified chamber. After washing, the sections were incubated with Cy3 goat anti-Mouse IgG (H + L) (Servicebio, GB21301), HRP conjugated goat anti-Rabbit IgG (H + L) (Servicebio, GB23303) or immunofluorescent TUNEL (Servicebio, G1501) reaction in a moist chamber (dark, 37°C, 1 h). The sections were then counterstained with DAPI (Servicebio, G1012) for the detection of nuclei. Finally, the stained sections were embedded in the resistance to fluorescence quenching sealing liquid and pictured using a fluorescence microscope (NIKON ECLIPSE C1, Japan).

### Cell Culture

Human renal mesangial cells (HRMCs) were obtained from ScienCell Research Laboratories, Santiago, CA, and cultured in Mesangial Cell Medium (MsCM, ScienCell Research Laboratories). HRMCs were plated on a poly-L-lysine coated flask (2 μg/cm^2^), and grown at 37°C in a humidified atmosphere containing 5% CO_2_. The cells in this experiment were used within 3–4 passages and were examined to ensure that they demonstrated the specific characteristics of mesangial cells. Mouse podocyte cell line MPC-5 was obtained from ATCC, Maryland, United States. The cells were grown on type I collagen in RPMI 1640 (10% FBS) with 50 U/ml IFN-γ at 33°C to 85% confluency and then transferred to 37°C without IFN-γ for 10–14 days for differentiation.

### Cell Viability and Proliferation Assay

Cell Counting Kit-8 (CCK-8) was used to measure cell proliferation and cell viability. Cells were seeded in each well of a 96-well culture plates (5 × 10^3^/well). After the treatment, 10 μl CCK-8 (Beyotime, Shanghai, China) was added and incubated for 1 h at 37°C. Absorbance was measured using a microplate reader (Thermo Fisher Sci-entific, Waltham, MA, United States) at a wavelength of 450 nm.

### Annexin V and Propidium Iodide Staining

Cells were plated and grown until they reached 60% confluence, and then treated with high glucose (50 mmol/L) or telmisartan. After 96 h, the collected cells were washed with cold PBS and resuspended in binding buffer. Annexin V-FITC and PI (eBioscience, Santiago, CA, United States) were added to the cellular suspension as in the manufacturer’s instructions, and a sample fluorescence of 10,000 cells was analyzed by flow cytometry conducted with FACScan (Becton, Dickinson and Company, Franklin, NJ, United States).

### Western Blotting

The renal cortex, HRMCs and MPC-5 were homogenized in Tissue or Cell Protein Extraction Reagent (Beyotime) supplemented with protease and phosphatase inhibitors (Merck, Whitehouse Station, NJ, United States). Samples were separated on a 10% SDS PAGE and transferred to nitrocellulose membrane (Pall Corporation, NY, United States). The membrane was blocked with 5% bovine serum albumin and blotted with antibody. Anti-PKCβ1 (Cell Signaling Technology, 46,809), anti-swiprosin-1 (Abcam, ab24368), TGF-β1 (Abcam, ab215715), Tubulin (Beyotime, AT819) and GAPDH (Beyotime, AF5009) were used at a concentration of 1:1,000. Proteins were visualized using an IRDye-conjugated anti-mouse or anti-rabbit secondary antibodys (Rockland, Limerick, PA, United States) at 1:5,000. Using ODYSSEY INFRARED IMAGING SYSTEM (LI-COR) to analyses the results.

### RNA Isolation and Real-Time RT-PCR

Total RNA was isolated from cells with TRIzol reagent (Invitrogen, Carlsbad, CA, United States) followed by chloroform isopropanol extraction and ethanol precipitation, and 1 μg of total RNA was reverse-transcribed using the PrimeScript RT reagent kit (Takara Bio, Otsu, Japan). RT-PCR was performed by the Thermal Cycler Dice Real Time System TP800 (Takara Bio, Otsu, Japan) by use of SYBR Green fluorescence signals. The following primers were used:

#### Human

PKCβ1, 5′-TTT​GAA​GGG​GAG​GAT​GAA​GAT​GAA-3′ (forward) and 5′-TGA​AGA​GTT​TAT​CAG​TGG​GGG​TCA​GTT​C-3′ (reverse);

AT_1_, 5′-ACC​TGG​CTA​TTG​TTC​ACC​CAA​T-3′ (forward) and 5′-TGC​AGG​TGA​CTT​TGG​CTA​CAA​G-3′ (reverse);

AT_2_, 5′-TAA​GCT​GAT​TTA​TGA​TAA​CTG​C-3′ (forward) and 5′-ATA​TTG​AAC​TGC​AGC​AAC​TC-3′ (reverse);

PPARγ, 5′-GAT​GCC​AGC​GAC​TTT​GAC​TC-3′ (forward) and 5′-ACC​CAC​GTC​ATC​TTC​AGG​GA-3′ (reverse);

GAPDH, 5′-CGG​AGT​CAA​CGG​ATT​TGG​TCG​TAT-3′ (forward) and 5′-AGC​CTT​CTC​CAT​GGT​GGT​GAA​GAC-3′ (reverse).

#### Mouse

PKCβ1, 5′-ATG​AGT​TCG​TCA​CGT​TCT​CCT-3′ (forward) and

5′-CCA​TAC​AGC​AGC​GAT​CCA​CAG-3′ (reverse);

AT_1_, 5′-TTG​TCC​ACC​CGA​TGA​AGT​CTC-3′ (forward) and

5′-AAA​AGC​GCA​AAC​AGT​GAT​ATT​GG-3′ (reverse);

AT_2_, 5′-CGC​AAC​TGG​CAC​CAA​TGA​G-3′ (forward) and 5′-AGG​GAG​GGT​AGC​CAA​AAG​GAG-3′ (reverse);

PPARγ, 5′-CTT​GGC​TGC​GCT​TAC​GAA​GA-3′ (forward) and 5′-GAA​AGC​TCG​TCC​ACG​TCA​GAC-3′ (reverse);

GAPDH, 5′-AAT​GGA​TTT​GGA​CGC​ATT​GGT-3′ (forward) and 5′-TTT​GCA​CTG​GTA​CGT​GTT​GAT-3′ (reverse).

PCR conditions were set as incubation at 95°C for 30 s followed by 40 cycles of 5 s at 95°C, 34 s at 64°C. Levels of mRNA were normalized with GAPDH and expressed as relative levels compared to control.

### Statistical Analyses

Data processing was analyzed by Origin 6.1 (OriginLab, Northampton, MA) and expressed as mean ± SD of at least three independent experiments. Statistical significance was determined using ANOVA. A value of *p* < 0.05 was considered statistically significant.

## Results

### Telmisartan Alleviated Early Renal Injury in STZ-Induced Type 1 Diabetes Rats

**FIGURE 1:**
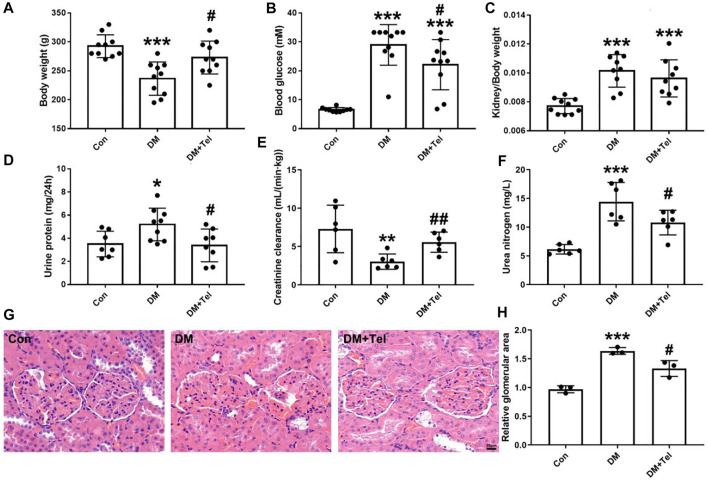
Telmisartan alleviated early renal injury in STZ-induced type 1 diabetes rats. Weight 180–200 g male rats were intraperitoneally treated with STZ (65 mg/kg body weight) to induce type 1 diabetes, and then received telmisartan (10 mg kg^−1^·d^−1^ po, group DM + Tel, *n* = 10) or vehicle (group DM, *n* = 10) by gavage for 4 weeks. The body weight **(A)**, blood glucose **(B)**, urine protein **(D)**, creatinine clearance **(E)** and urine nitrogen **(F)** were measured 24 h before anesthesia. Removed kidneys for kidney-to-body weight ratio **(C)**, histological imaging **(G)** and relative glomerular area **(H)** were shown. Data represent the mean ± SD and were analysed with one-way ANOVA (*p < 0.05, **p < 0.01, ***p < 0.001 *vs*. Con group; #*p* < 0.05, ##*p* < 0.01 *vs*. DM group). Scale bar = 20 μm. Con, control; DM, diabetes mellitus; DM + Tel, diabetes mellitus with telmisartan treatment.

STZ-injected rats produced characteristic symptoms of diabetes at the 4th weeks, including declined body weight gain (Figure 1A) hyperglycemia (Figure 1B), increased kidney-to-body weight ratio, 24-h urine protein and urea nitrogen (Figures 1C,D,F), and decreased creatinine clearance rate (Figure 1E). Telmisartan treatment significantly increased body weight (Figure 1A) and creatinine clearance rate (Figure 1E), decreased blood glucose (Figure 1B), urine protein (Figure 1D) and urea nitrogen (Figure 1F). Although the kidney-to-body weight ratio of the telmisartan treatment group was reduced, there was no significant difference between these two groups (Figure 1C). Histological analysis showed that extracellular matrix deposition (Figure 1G) and glomerular volume (Figure 1H) were amplified in diabetic rats, while telmisartan attenuated extracellular matrix deposition significantly (Figure 1G).

**FIGURE 2:**
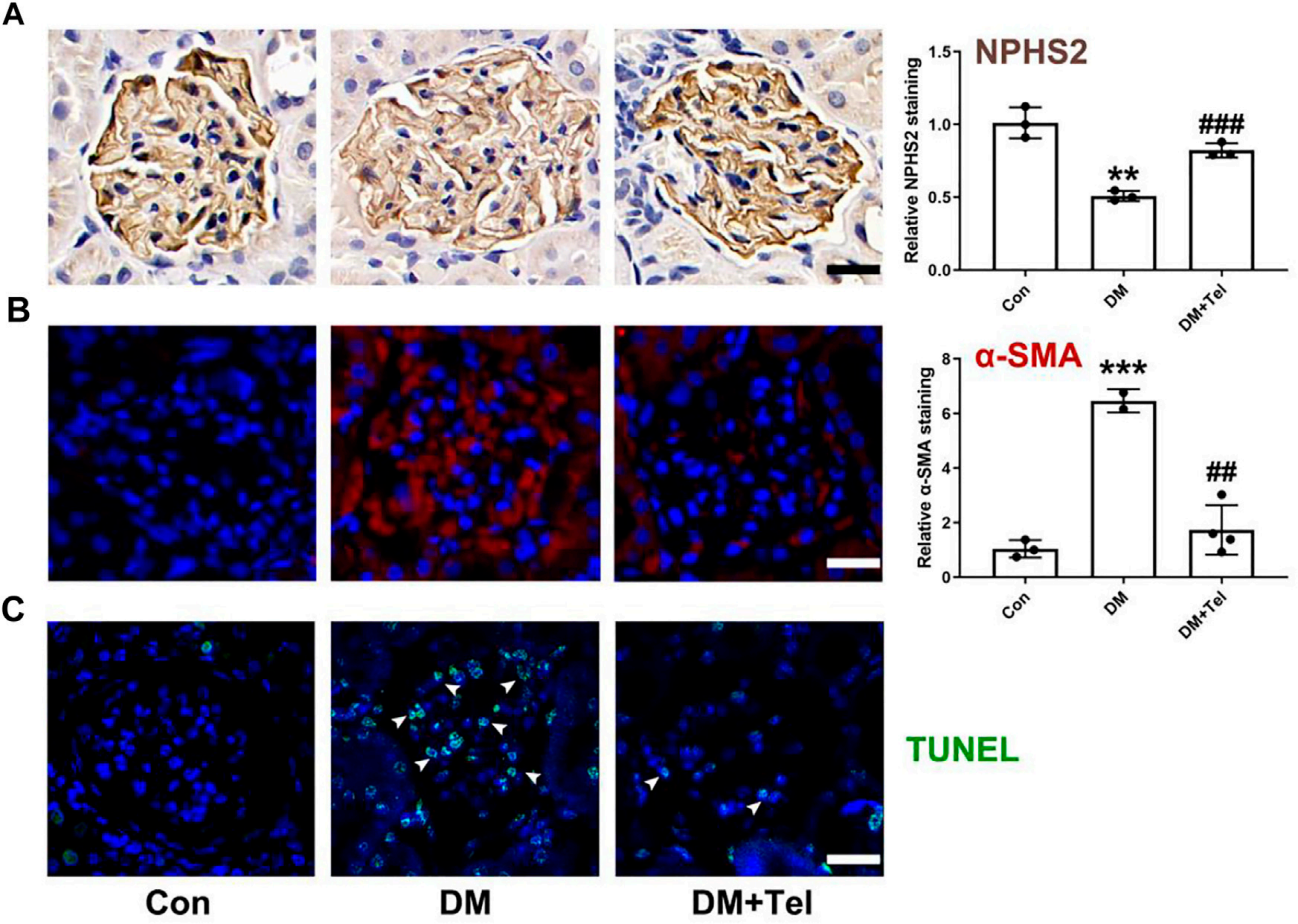
Telmisartan decreased expression of NPHS2, α-SMA and cell apoptosis in diabetic glomeruli. **(A)** NPHS2 staining, **(B)** α-SMA staining, and **(C)** TUNEL staining were used to observed podocyte loss, mesangial matrix expansion and cell apoptosis in glomeruli of early diabetic rats. Data represent the mean ± SD and were analysed with one-way ANOVA (**p < 0.01, ***p < 0.001 *vs*. Con group; ##*p* < 0.01, ###*p* < 0.001 *vs*. DM group). Scale bar = 20 μm. Con, control; DM, diabetes mellitus; DM + Tel, diabetes mellitus with telmisartan treatment.

To further estimate the effect of telmisartan on early glomerular damage of diabetic rats, we observed the changes in podocytes loss and mesangial matrix expansion. NPHS2, also known as podocin, is a characteristic protein molecule located on the slit diaphragm of podocytes. The decreased expression of NPHS2 in diabetic glomeruli represents the damage and loss of podocytes (Tanoue et al., 2021), and telmisartan could alleviate the down-regulation of NPHS2 (Figure 2A). α-SMA, an indicator of the activation of mesangial cells under hyperglycemia to secrete extracellular matrix, could be inhibited after telmisartan treatment (Figure 2B). In addition, telmisartan reduced the number of apoptotic cells in the glomeruli of early diabetic rats (Figure 2C).

### Telmisartan Alleviated the Decreased Cell Viability of Podocytes but Not Mesangial Cells Induced by High Glucose *In Vitro*

**FIGURE 3:**
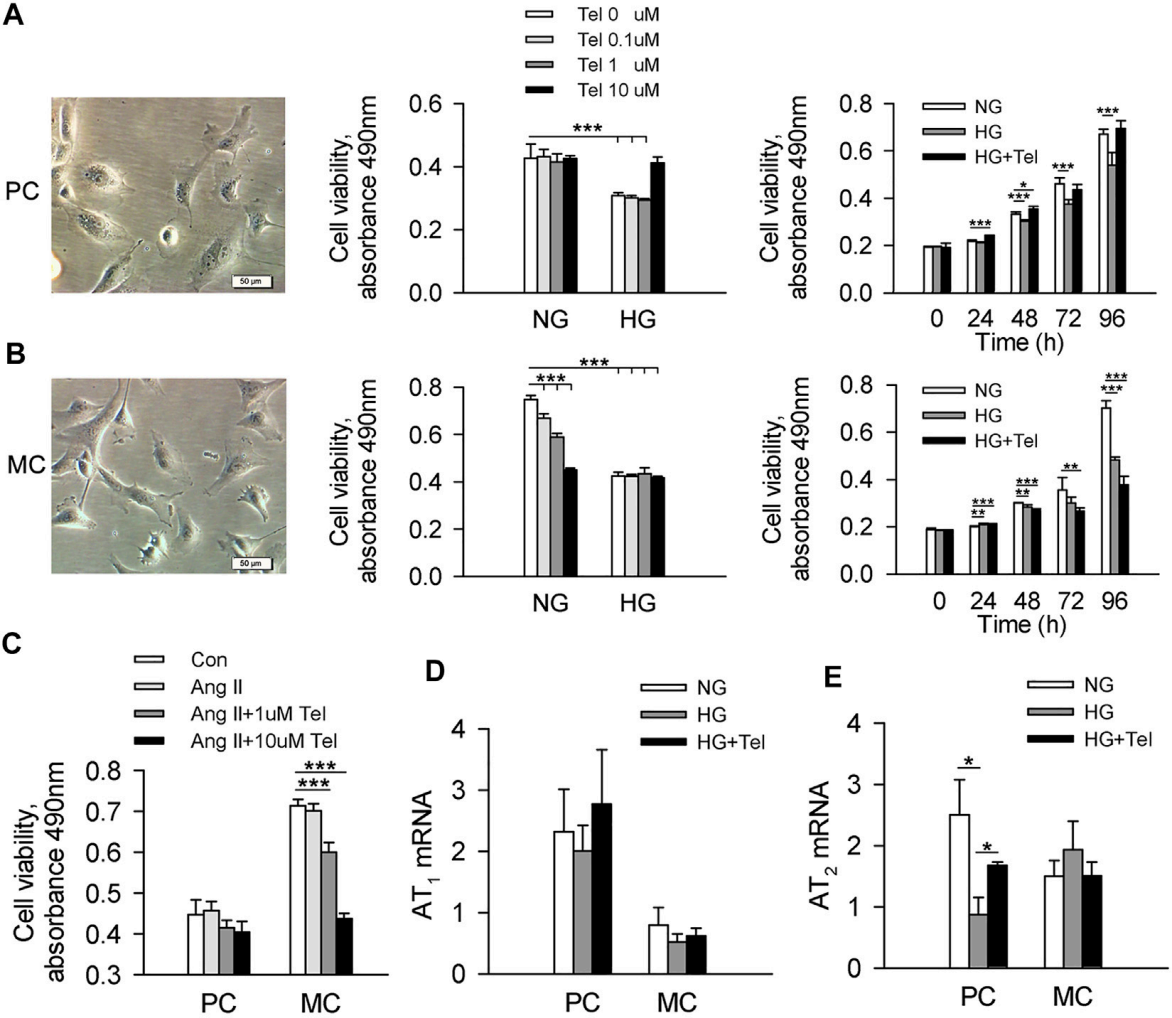
Telmisartan alleviated the decreased cell viability of podocytes but not mesangial cells induced by high glucose *in vitro*. **(A)** Representative images of mouse podocyte cell (MPC-5) lines. And the effect of telmisartan on MPC-5 cell viability in normal glucose (5.5 mmol) or high-glucose (HG, 50 mmol/L) environment were measured. **(B)** Representative images of human renal mesangial cells (HRMCs). And the effect of telmisartan on HRMCs cell viability in normal glucose (5.5 mmol) or high-glucose (HG, 50 mmol/L) environment were measured. **(C)** Cell viability of podocytes and mesangial cells with HG or telmisartan treatment. The mRNA expression of AT1 **(D)** and AT2 **(E)** receptor in podocytes and mesangial cells treated with telmisartan (10 μM) in a high-glucose environment. Data are shown as mean ± SD of *n* = 3–5 (*p < 0.05, **p < 0.01, ***p < 0.001). PC, mouse podocyte cell lines; MC, Human renal mesangial cells; NG, normal glucose; HG, high glucose; HG + Tel, high glucose mellitus with telmisartan treatment; Con, control; Ang II, angiotensin II; Ang II + Tel, angiotensin II unite with telmisartan treatment.

To reveal the different effects of telmisartan on podocytes and mesangial cells, we used human renal mesangial cells (HRMCs) and mouse podocyte cell (MPC-5) lines to investigate the effect of telmisartan on cell viability in a high-glucose (HG, 50 mmol/L) environment, respectively. As the results showed, cell viability of MPC-5 was significantly decreased when stimulated with HG after 96 h, while telmisartan could restore cell viability at the concentration of 10 μM. The cell viability of MPC-5 began to decrease significantly after 48 h of HG stimulation, while telmisartan (10 μM) could fully restore this decrease at three time points of 48, 72 and 96 h (Figure 3A). Surprisingly, cell viability of HRMCs was significantly decreased when stimulated with telmisartan dose-dependently even in a normal glucose medium. Telmisartan also could not improve the decreased viability of HRMCs induced by HG (Figure 3B). Furthermore, we investigated whether the effect of telmisartan on podocytes and mesangial cells was related to its known target angiotensin II receptor. Here, angiotensin II did not reduce cells viability, and telmisartan also only damaged the viability of mesangial cells (Figure 3C). Meanwhile, HG and telmisartan did not affect AT_1_ receptor mRNA expression in these 2 cell lines (Figure 3D). Telmisartan is highly selective for AT_1_ receptor, while AT_2_ receptor may be activated compensatory by angiotensin II. As the results shown, HG-induced down-regulation of AT_2_ receptor in podocytes could be inhibited by telmisartan. However, the mRNA expression of AT_2_ receptor in mesangial cells was not affected by HG or telmisartan (Figure 3E).

### Telmisartan Alleviated Podocytes Apoptosis Induced by High Glucose *In Vitro*

**FIGURE 4:**
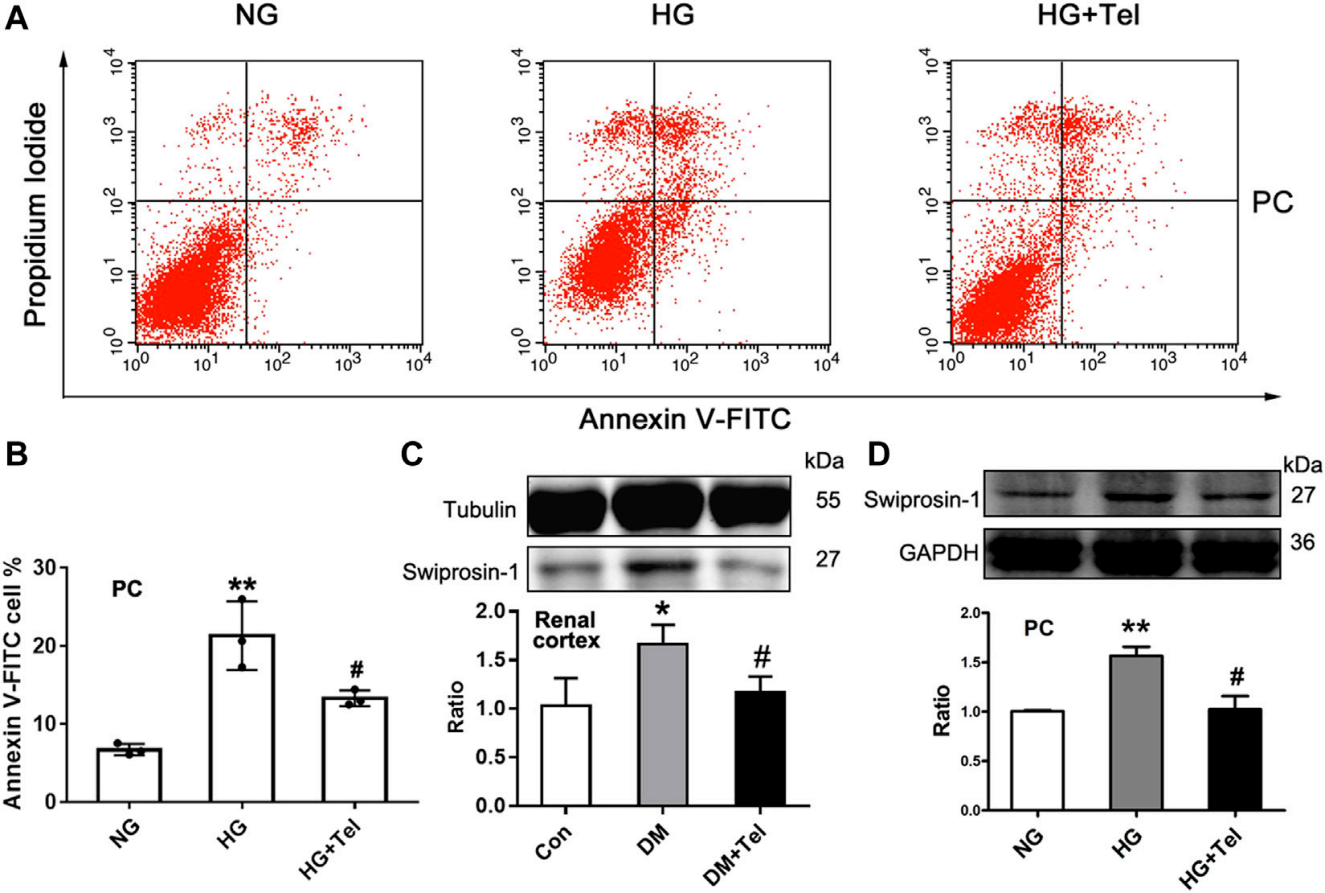
Telmisartan alleviated podocyte apoptosis induced by high glucose *in vitro*. **(A)** Flow cytometry analysis of podocytes apoptosis. **(B)** The statistical results of apoptosis in MPC-5 incubated with normal glucose (5.5 mmol) or high-glucose (HG, 50 mmol/L) environment with or without telmisartan (10 μM) for 96 h. **(C)** Immunoblot of swiprosin-1 expression in diabetic renal cortex with or without telmisartan treatment. **(D)** Swiprosin-1 expression of MPC-5 incubated in normal glucose (5.5 mmol) or high-glucose (HG, 50 mmol/L) environment with or without telmisartan (10 μM) for 96 h. Representative blots of three independent experiments are shown. Data are shown as mean ± SD of n = 3 (*p < 0.05, **p < 0.01 *vs*. NG or Con group; #*p* < 0.05 *vs*. HG or DM group). PC, mouse podocyte cell lines; NG, normal glucose; HG, high glucose; HG + Tel, high glucose mellitus with telmisartan treatment; Con, control; DM, diabetes mellitus; DM + Tel, diabetes mellitus with telmisartan treatment.

As telmisartan could reduce glomerular cell apoptosis in diabetic rats and restore the decreased viability of podocytes induced by HG, we further confirmed that telmisartan attenuated MPC-5 apoptosis induced by HG *in vitro via* measuring the ratio of apoptotic Anexin-V and IP-stained cells by cytometry (Figures 4A,B). We have previously reported that swiprosin-1 participates in the apoptosis of podocytes in early diabetic kidney injury (Wang et al., 2018b). Here, we also found that telmisartan decreased the expression of swiprosin-1 in diabetic renal cortex (Figure 4C) and HG-stimulated MPC-5 (Figure 4D).

### Telmisartan Induced Apoptosis of Mesangial Cells With or Without HG Stimulation *In Vitro*

**FIGURE 5:**
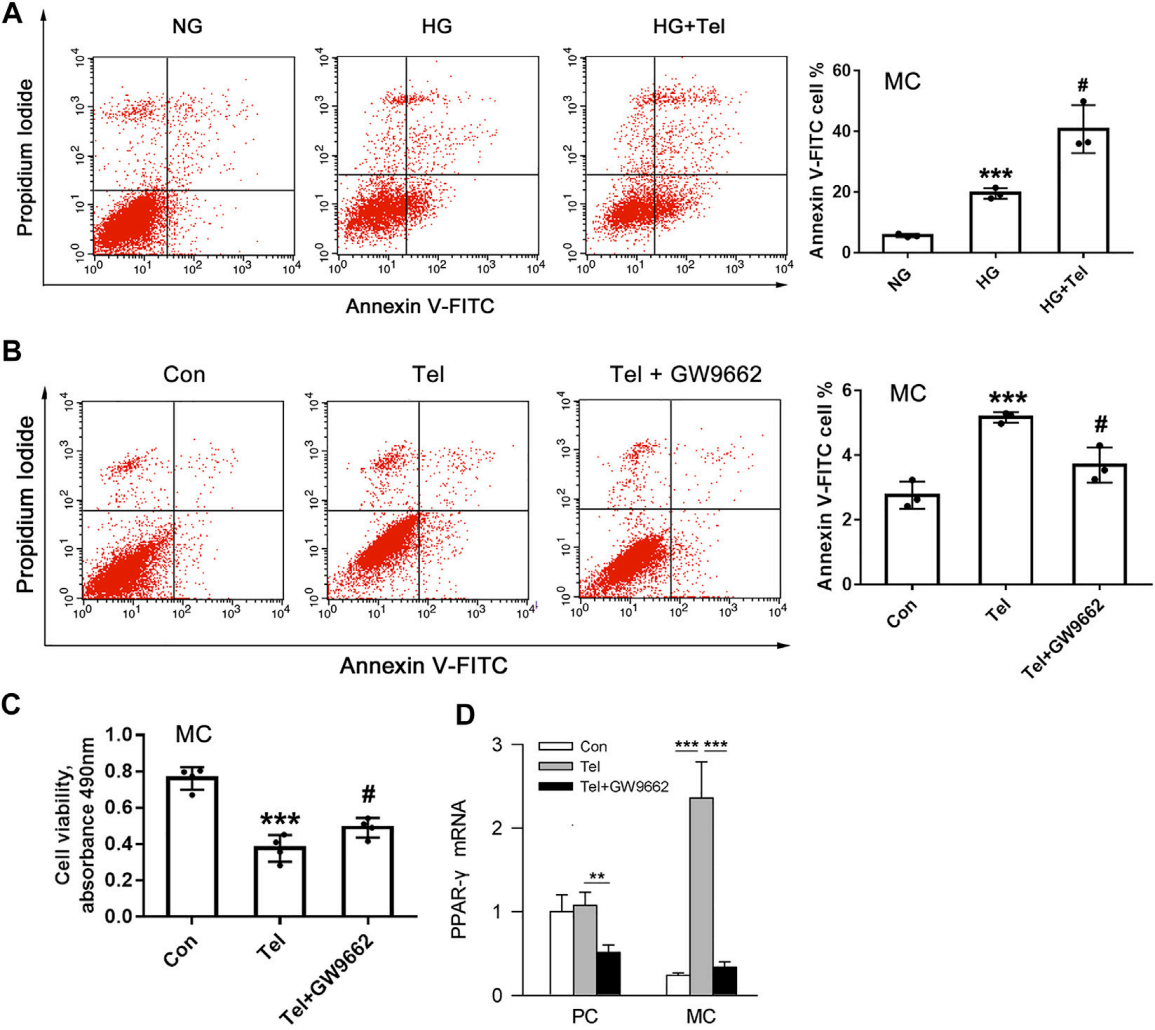
Telmisartan induced apoptosis of mesangial cells with or without HG stimulation *in vitro* **. (A)** Flow cytometry analysis of human renal mesangial cells apoptosis with HG or telmisartan treatment for 96 h. **(B)** Representative images of flow cytometry apoptosis analysis in HRMCs with telmisartan (10 μM) or GW9662 (10 μM) treatment for 96 h. **(C)** Cell viability of human renal mesangial cells with telmisartan (10 μM) or GW9662 (10 μM) treatment for 96 h. **(D)** The effect of telmisartan or GW9662 on PPAR-γ mRNA expression in podocytes and mesangial cells were measured. Data are shown as mean ± SD of *n* = 3 (***p < 0.001 *vs*. NG or Con group; #*p* < 0.05 *vs*. HG or Tel group). PC, mouse podocyte cell lines; MC, Human renal mesangial cells; NG, normal glucose; HG, high glucose; Con, control; Tel, telmisartan; GW9662, PPAR-γ blocker; GW9662 + Tel, PPAR-γ blocker unite with telmisartan treatment.

The above results have shown that telmisartan reduces glomerular α-SMA and directly inhibits the cell viability of HRMCs. We further investigated whether telmisartan itself could induce mesangial cells apoptosis. Under HG stimulation, telmisartan could further increase the apoptosis of HRMCs significantly (Figure 5A). Besides, telmisartan itself could also directly induce apoptosis of HRMCs in normal glucose medium (Figure 5B). PPARγ, as another target that could be activated by telmisartan, is widely involved in the apoptosis of various cells (Ayza et al., 2020). Telmisartan-induced HRMCs apoptosis and cell viability damage were significantly inhibited by the PPARγ blocker GW9662 (Figures 5B,C). Interestingly, telmisartan specifically induced high expression of PPARγ in HRMCs but not in MPC-5 (Figure 5D).

### Telmisartan Decreased PKCβ1 and TGFβ1 in Mesangial Cells

**FIGURE 6:**
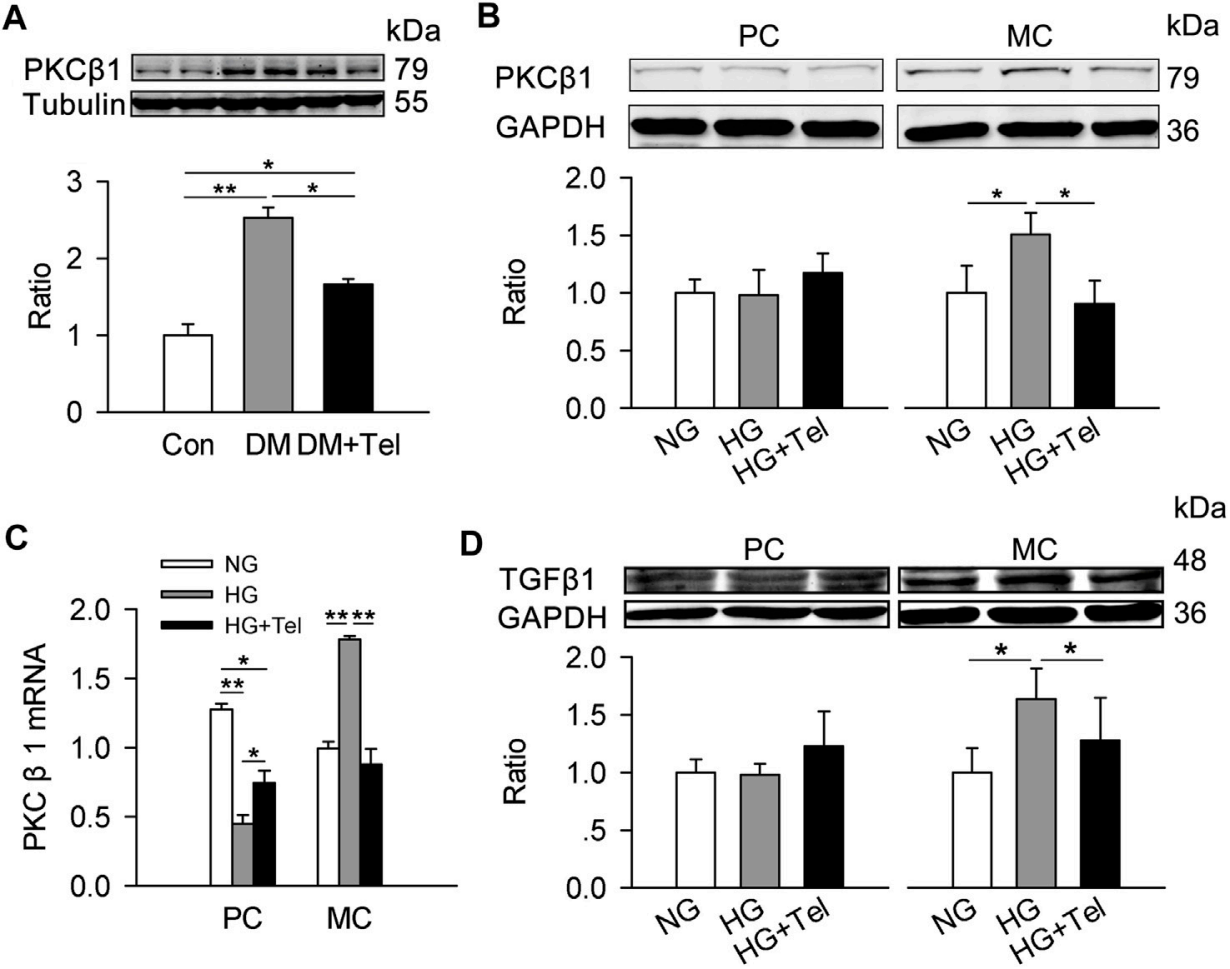
Telmisartan decreased PKCβ1 and TGFβ1 in mesangial cells. **(A)** Immunoblot of PKCβ1 expression in diabetic renal cortex with or without telmisartan treatment. **(B)** PKCβ1 expression of podocytes and mesangial cells incubated in normal glucose (5.5 mmol) or high-glucose (HG, 50 mmol/L) environment with or without telmisartan (10 μM) for 96 h. **(C)** The mRNA expression of PKCβ1 in podocytes and mesangial cells incubated with normal glucose (5.5 mmol) or high-glucose (HG, 50 mmol/L) environment with or without telmisartan (10 μM) for 96 h. **(D)** The effect of normal glucose (5.5 mmol) or high-glucose (HG, 50 mmol/L) with or without telmisartan treatment on TGFβ1 expression in podocytes and mesangial cells were measured. Representative blots of two independent experiments are shown (*p < 0.05, **p < 0.01). PC, mouse podocyte cell lines; MC, Human renal mesangial cells; NG, normal glucose; HG, high glucose; HG + Tel, high glucose mellitus with telmisartan treatment; Con, control; DM, diabetes mellitus; DM + Tel, diabetes mellitus with telmisartan treatment.

TGF-β is a major mediator of matrix expansion in diabetic glomerulus and its up-regulation stimulated by HG in mesangial cells requires PKCβ1 (Wu et al., 2009). As the results showed below/above, telmisartan inhibited the up-regulation of PKCβ1 in renal cortex of diabetic rats (Figure 6A). Specifically, HG induced the protein expression of PKCβ1 in HRMCs but not in MPC-5, and telmisartan could reduce PKCβ1 expression induced by HG in HRMCs (Figure 6B). The mRNA expression of PKCβ1 induced by HG was also suppressed by telmisartan in HRMCs (Figure 6C). Reversely, HG down-regulated the mRNA expression of PKCβ1 in MPC-5, which could be partially restored by telmisartan (Figure 6C). Similarly, HG specially induced TGFβ1 expression in HRMCs but not MPC-5, and telmisartan significantly inhibited HG-induced TGFβ1 in HRMCs (Figure 6D).

## Discussion

Telmisartan is a selective AT_1_ receptor blocker which has been used clinically for reducing elevated blood pressure and urinary protein excretion in hypertensive patients (Baden et al., 2008; Mann et al., 2009). Several clinical trials have suggested that telmisartan is effective to reduce proteinuria in patients with macroalbuminuria, and delay the onset and progression of diabetic nephropathy (Makino et al., 2005; Nakamura et al., 2010; Fujita et al., 2011; Schmieder et al., 2011). In the present study, oral treatment with telmisartan in STZ-induced diabetes rats prevented the onset of early abnormalities in renal and overall including the decrease in body weight, blood glucose and urine protein. These results confirmed that telmisartan has renoprotection in early stage diabetic nephropathy mice. More importantly, this study found that the protective effect of telmisartan on diabetic glomeruli was reflected in the anti-apoptotic and pro-apoptotic effects on podocytes and mesangial cells, respectively.

Advanced glycation end products (AGE) could cause podocyte DNA injury and detachment partly through stimulation of Ang II-AT_1_R axis, thus supplying a innovative beneficial feature of telmisartan in DKD (Fukami et al., 2013). In normotensive, low-grade proteinuric glomerular diseases, treatment with telmisartan in the early stage of disease, attenuates glomerular and tubulointerstitial damage (Villa et al., 2011). And several pathways probably linked to the pleiotropic consequences including growth factor signaling, mammalian target of rapamycin signaling, protein ubiquitination, the Wnt-beta catenin pathway and hypoxia signaling (Villa et al., 2011).

Recently, we reported that swiprosin-1 (Wang et al., 2018b), another name as EF hand domain containing 2 (EFhd2), that played a critical part in the progression of DKD initiated after the induction, while it located in podocytes of the mouse glomerulus. Swiprosin-1 absence ameliorated mitochondria-dependent podocytes apoptosis stimulated by hyperglycemia or high-glucose through p38 MAPK signaling pathway. Here, we also found that telmisartan inhibited hyperglycemia or high-glucose induced expression of swiprosin-1 both *in vivo* and *in vitro*, which indicated the anti-apoptosis effect of telmisartan on podocytes may be related to the regulation of swiprosin-1 expression.

Mesangial cells proliferation and excessive deposition of extracellular matrix proteins has been ascertained contributing to the development of DKD (Lee et al., 2004). Previous studies showed that high glucose could induce expression of mesangial extracellular matrix proteins under hyperglycemia (Taniguchi et al., 2013). α-SMA is generally used to differentiate mesangial cells from other glomerular cells in STZ-induced diabetes mice, and increased α-SMA expression could be as the marker of mesangial cells phenotypic shifts from the non-activated phase to the proliferative, secretory activated phase (Niu et al., 2014). Here, we found that telmisartan decreased α-SMA expression in diabetic glomerulus. In addition, it has been reported that mesangial cells proliferation has a significant impact on the pathogenesis of DKD (Zeng et al., 2013). Our results in this study were reflecting the time- and dose-dependent depressed effect of telmisartan on mesangial cells proliferation related with pro-apoptotic characteristic.

Clinical evidence recommends that telmisartan is more efficient than losartan in ameliorating proteinuria in hypertensive person with DKD, which may be related to its ability to partially agonize PPARγ (Bichu et al., 2009). Furthermore, these beneficial changes such as the prevention of renal atrophy and fibrosis of telmisartan were connected with a diminishing in the expression of TGFβ1 and other proinflammatory and profibrotic cytokine genes *via* the PPARγ/HGF activation (Kusunoki et al., 2012), independent of Ang II type 1 receptor blockade. Here, we also found telmisartan specifically activated PPARγ gene expression in mesangial cells, and pro-apoptotic effect caused by telmisartan to mesangial cells could be alleviated by PPARγ inhibitors.

PKCβ1 is one of the extensively expressed family of serine–threonine kinases that transduce a wide range of cellular progressions by substrate-specific phosphorylation (Newton, 1995). It has been reported that not only increased PKCβ activity but also its mRNA levels are observed in human diabetic nephropathy biopsies (Langham et al., 2008). Hyperglycemia-induced PKCβ expression and activation has pleiotropic effects in mesangial cells, including the promoting excessive accumulation of ECM proteins (Brownlee, 2001). Studies have shown that inhibition of PKCβ attenuates glomerular hypercellularity and extracellular matrix expansion in db/db mice and glomerular dysfunction in STZ-rats (Ishii et al., 1996; Koya et al., 2000). Likewise, PKCβ inhibitor attenuated platelet derived growth factor (PDGF)-driven mesangial cell proliferation and collagen production (Tokuyama et al., 2011). In our study, telmisartan reduced the upregulation of PKCβ1 mRNA and protein expression in hyperglycemia-stimulated mesangial cells. In addition, TGFβ1 expression in mesangial cells induced by high glucose could also be inhibited by telmisartan.

Both AT_1_ and AT_2_ receptors, well known as seven transmembrane spanning G protein-coupled receptors, have been cloned and pharmacologically illustrated (Touyz and Berry, 2002). The AT_1_ receptors can be selectively antagonized by telmisartan, while AT_1_ receptor blocker can induce the expression of AT_2_ receptors (Touyz and Berry, 2002). Studies has shown that AT_1_ receptors exert their influences by restraining cell growth, and by provoking apoptosis (Horiuchi et al., 1997; Touyz et al., 1999). Moreover, AT_2_ receptors induce cell apoptosis in a specific conformation though p38 MAPK-mediated apoptotic signaling (Miura and Karnik, 2000). In our present paper, expression of AT_1_ and AT_2_ mRNA was unchanged in cultured mesangial cells stimulated with telmisartan or hyperglycemia. Therefore, telmisartan-induced mesangial cells apoptosis and decreased expression of PKCβ1 might not mediated by AT_1_ and AT_2_ receptors.

In conclusion, telmisartan attenuated early glomerular injury in type 1 diabetic rats by inhibiting podocyte apoptosis and promoting mesangial apoptosis. The anti-apoptotic effect of telmisartan in podocytes may be related to its inhibition of swiprosin-1 expression, meanwhile the pro-apoptotic effect on mesangial cells was partially associated with its agonistic effect on PPARγ. Additionally, telmisartan selectively blocked the expression of PKCβ1/TGFβ1 in mesangial cells but not in podocytes. Advanced studies are necessitated to elucidate the opposite but beneficial effects of telmisartan on podocytes and mesangial cells and the underlying molecular mechanisms.

## Data Availability

The original contributions presented in the study are included in the article/Supplementary Material, further inquiries can be directed to the corresponding authors.
